# The Feasibility of Surface Electromyography in Monitoring Orbicularis Oculi Recovery after Anterior Approach Levator Aponeurosis Advancement

**DOI:** 10.3390/jcm11030731

**Published:** 2022-01-29

**Authors:** Larysa Krajewska-Węglewicz, Marta Banach, Ewa Filipiak, Joanna Sempińska-Szewczyk, Piotr Skopiński, Małgorzata Dorobek

**Affiliations:** 1 Department of Ophthalmology, Central Clinical Hospital of the Ministry of Interior and Administration in Warsaw, 02-507 Warsaw, Poland; ewa.filipiak@cskmswia.gov.pl (E.F.); joanna.sempinska@cskmswia.gov.pl (J.S.-S.); 2 Department of Neurology, Central Clinical Hospital of the Ministry of Interior and Administration in Warsaw, 02-507 Warsaw, Poland; martabanach@yahoo.com (M.B.); malgorzata.dorobek@cskmswia.gov.pl (M.D.); 3 Department of Neurology, Jagiellonian University Medical College, 31-008 Krakow, Poland; 4 Department of Histology and Embryology, Medical University of Warsaw, 02-091 Warsaw, Poland; pskopin@wp.pl; 5 Department of Applied Physiology, Mossakowski Medical Research Institute, Polish Academy of Sciences, 00-901 Warsaw, Poland

**Keywords:** surface electromyography, ptosis, orbicularis oculi, eyelid

## Abstract

Introduction: In this article, we propose a new application for eyelid surface electromyography (sEMG). By placing the electrode in the mid-pretarsal area of the upper eyelid, one can easily perform a fast examination and achieve repeatable results. We believe that this technique may increase the feasibility of eyelid sEMG in clinical practice. Methods: 126 sEMG examinations of the upper eyelid were performed by using the above-described method. Thirty-nine controls and 29 ptotic patients were enrolled. The controls underwent one measurement while the ptotic patients were employed for four sessions: Before anterior approach levator aponeurosis advancement (LAA), 2 weeks, 3 months, and more than 6 months after surgery. The relaxation and maximal contraction of the orbicularis oculi muscle (OOM) using root mean square (RMS) values were measured. Results: The results showed a statistically significant decrease in RMS values of the maximal contraction of the OOM 2 weeks after surgery (*p* < 0.05) and 3 months after surgery (*p* = 0.03). Six months postoperatively, there were no statistically significant differences in OOM activity compared to preoperative values (*p* = 0.2). Conclusions: Eyelid sEMG may be a useful diagnostic tool in post-operative OOM recovery monitoring. sEMG parameters of the maximal contraction of the OOM normalize within 6 months after anterior approach LAA. Electrode placement in the mid-pretarsal area of the upper eyelid offers several advantages and therefore may enhance the feasibility of sEMG in clinical practice.

## 1. Introduction

Many of the anterior-approach oculoplastic procedures require an incision or resection of the orbicularis oculi muscle (OOM). Levator aponeurosis advancement (LAA) is one of the most frequently performed methods. LAA can be performed from the anterior or posterior approach [1,2]. In the anterior approach LAA, a strip of OOM is usually excised [3]. Resecting the OOM in the anterior approach LAA may lead to lagophthalmos. Moreover, it has not been established whether the OOM fully recovers from the anterior-approach ptosis surgery.

Surface EMG (sEMG) is a noninvasive, quantitative, and objective method of assessing muscle activity. It allows the measurement of muscle activity in both eyelids simultaneously. The utility of sEMG in the assessment of OOM, mainly in facial palsy, has been proven in experimental studies [4,5,6]. However, there are still limitations in the detection and characterization of the signal. One of the main concerns is crosstalk with adjacent muscles. The electrode placement seems to play a critical role in this artifact. To date, only a few studies on sEMG of the eyelid [4] or facial [7] muscles activity in healthy individuals have been conducted. Developing a reliable examination technique and obtaining information representative of muscle activation may lead to using sEMG in OOM disorders, assessing the results of eyelid surgery, and rehabilitation in paralysis.

### The Anatomical Aspects of Cross-Talk Limitation

The OOM is a striated muscle that lies just below the skin. It is a large muscle, considering eyelid structures. Anatomically, it is divided into four parts: Orbital, preseptal and pretarsal segments, and the muscle of Riolan. The orbital part overlies the bony orbital rim. The palpebral portion is positioned over the mobile eyelid and is divided into preseptal and pretarsal parts. The preseptal part overlies the orbital septum in both upper and lower eyelids. The pretarsal OOM is positioned over the tarsal plates. Between the tarsal plate and the pretarsal OOM runs the muscle of Riolan. Below the OOM lies the orbital septum that separates the preaponeurotic fat pads [8]. The location of the OOM directly below the skin and above the fat pads and the septum facilitates the sEMG examination due to the short distance between the muscle and the electrode and the isolation from other structures situated below. In addition, the rigid tarsal plate limits skin-fold formation during OOM contraction, which helps prevent electrode disconnection.

The frontalis muscle is a large and strong paired muscle that extends from the coronal suture line towards the supraorbital rim. Its medial fibers interdigitate with those of the procerus muscle. Under the brow, frontalis muscle fibers blend with the corrugator muscle and the depressor supercilii. The sEMG signal from the palpebral or supraorbital area of the OOM is compromised by the activation of the above-mentioned muscles.

OOM in the lower eyelid neighbors the zygomaticus major, zygomaticus minor, levator labii superioris, and levator labii superioris alaque nasi [9]. These large muscles interfere with OOM during contraction and are a source of crosstalk.

Taking into consideration these anatomical conditions, we assumed that the pretarsal area represents less crosstalk than any other part of the OOM.

We propose a new application for eyelid sEMG using single-electrode placement in the pretarsal area of the upper eyelid. Using RMS analysis of the maximal contraction of the OOM, the objectives of our study included (1) implementing this technique in a clinical setting in which patients presented with involutional ptosis; (2) comparing results in healthy controls to patients with involutional ptosis; (3) comparing results in patients with ptosis before and after surgery and (4) assessing the feasibility of sEMG in muscle recovery after ptosis surgery.

## 2. Materials and Methods

### 2.1. Participants

The prospective experimental study was conducted in the Department of Ophthalmology and the Department of Neurology at the Central Clinical Hospital of the Ministry of Interior and Administration in Warsaw. Sixty-eight participants (39 controls and 29 patients with involutional ptosis) were included in the study. The exclusion criteria were previous eyelid surgery or injury, eyelid retraction, lagophthalmos, eyelid skin lesions, stroke, myasthenia gravis, myopathies, and lack of consent.

### 2.2. Study Implementation

The study was implemented in the Electromyography Unit of the Department of Neurology. A Keypoint electromyography device with a 24kHz resolution and the Keypoint-Net Program was applied (manufactured by Medtronic A/S Skovlunde, Denmark). For standard BR assessment, the sweep speed of the EMG device was set at a 50–800 ms/division interval according to the interstimulus interval. Amplitude sensitivity was set at s 100–200 uV/division, and upper and lower frequency filters were set between 20 Hz and 10 kHz.

**Figure 1:**
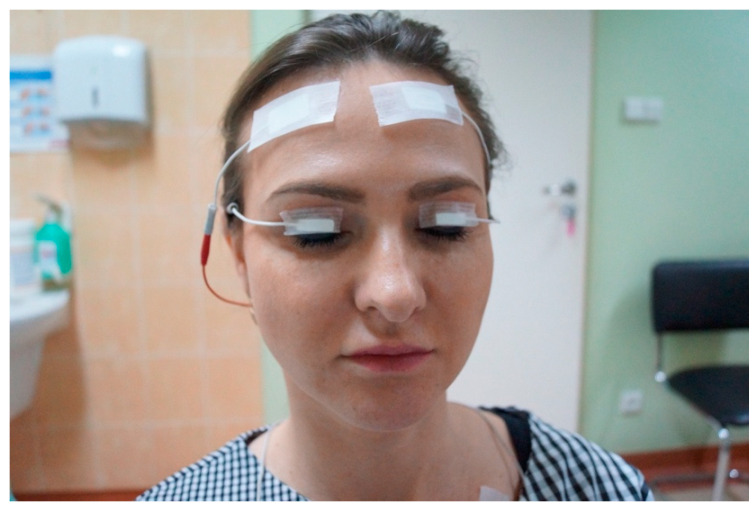
Electrode placement in the middle of the upper eyelid, 5 mm above eyelid margin, secured with adhesive tape.

During the examination, the subject was in a sitting position with their head in an upright position. The active electrode was placed in the central portion of the upper eyelid horizontally, 5 mm above the lash line, while the reference electrode was placed on the forehead (Figure 1). The ground electrode was placed on the left clavicular area. To eliminate factors reducing any skin resistance to the sEMG measuring signals, the measurement site was cleaned with medical alcohol cotton and its surfaces were to be completely dried before the electrode was placed. The cables were immobilized with tape. The dimensions of each silver-chloride active electrode were 5 × 8 mm. The electrodes were placed using conductive gel and adhesive tape.

**Figure 2:**
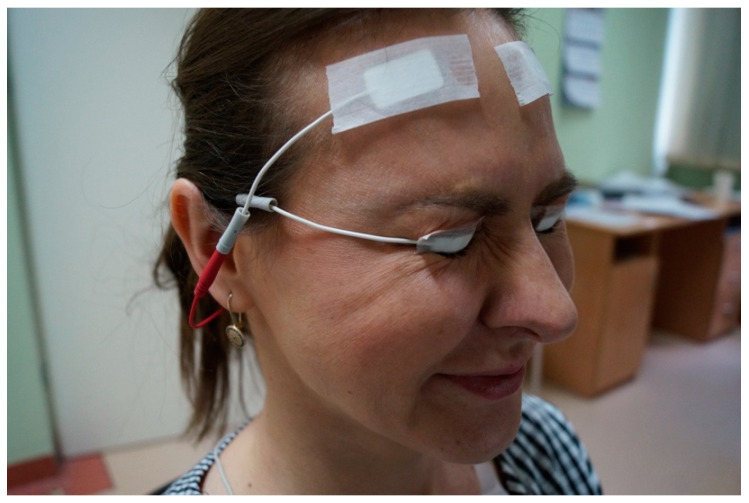
Subject performing maximal contraction of the OOM.

The sEMG examination was standardized and performed by one technician in the presence of the ophthalmologist (LKW). The procedure was explained to the participants. The real measurement was conducted when a subject fully learned how to perform the movement after preliminary measurement. The controls were committed to one research session lasting approximately 20 min. This included informed consent, setup time, and cleanup time. The ptotic patients were employed for four sessions (before surgery, 2 weeks after, 3 months, and more than 6 months after surgery). A one-time electromyography signal measurement was set at five seconds. Each test was repeated with three measurements. The average value of all three measurements was used as a measurement value. Between each measurement, subjects were required to take a one-minute rest. The measurement was taken while performing maximum eyelid closure (Figure 2) and with eyes open in the primary gaze. This represents the maximal tension and relaxation of the OOM, respectively. Raw sEMG signals were rectified and then polished using a Root Mean Square algorithm (RMS).

### 2.3. Ptosis Repair

All patients underwent anterior approach LAA. When indicated, blepharoplasty was performed. A strip of OOM was excised in all cases. After LAA, OOM was closed with Vicryl 6.0 interrupted sutures. The skin incision was closed with a running Prolene 5.0 suture.

### 2.4. Statistical Analysis

Statistical analysis was performed. The Shapiro–Wilk test was used to test the normal or non-normal distribution of variables. The mean and standard deviations, as central tendency measures, were estimated in the normal distributed variables, and the median and interquartile range were estimated in the not normally distributed variables.

The nonparametric Mann–Whitney U-test was adopted to evaluate whether there was a statistically significant difference in the average contraction values between controls and patients. Meanwhile, the comparison of the average contraction values between patients before and after surgery was analyzed using the Wilcoxon test. A level of *p* < 0.05 was selected as the threshold for the statistical significance.

## 3. Results

The experiment was well tolerated by all participants. Demographics of the examined groups are summarized in Table 1.

In the control group, 39 individuals had a single sEMG examination. Patients in the ptosis group initially consisted of 29 participants prior to surgery, 28 people 2 weeks after the operation, 13 patients after 3 months, and 17 patients more than 6 months after the surgery. That provided 87 sEMG examinations in the patient group and 126 measurements in both groups. An equal number of left and right eyelids were operated on.

**Figure 3:**
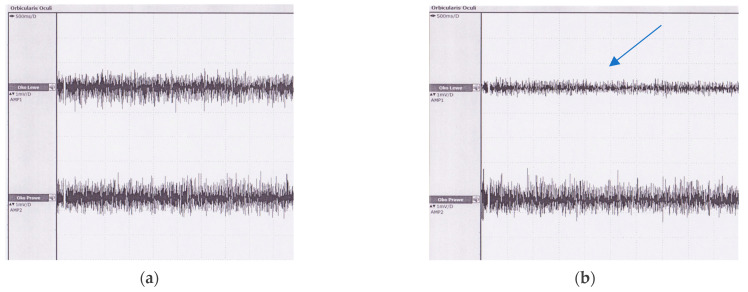
Raw data of the maximal contraction of OOM before surgery (**a**) and 2 weeks after (**b**). Significant decrement in the amplitude of OOM activation in the left eyelid after surgery is presented (arrow).

Figure 3 presents an example of the sEMG signal registered during maximal contraction of the OOM recorded in one patient before and two weeks after surgery.

Maximal contraction and relaxation of the OOM were similar in both controls and patients prior to surgery (*p* > 0.05). None of the subjects experienced any limitation in the maximal contraction of the eyelid with an electrode attached in the mid-pretarsal area of the upper eyelid. The results of RMS values in men and women and in left and right eyelids were compared (Table 2).

**Figure 4:**
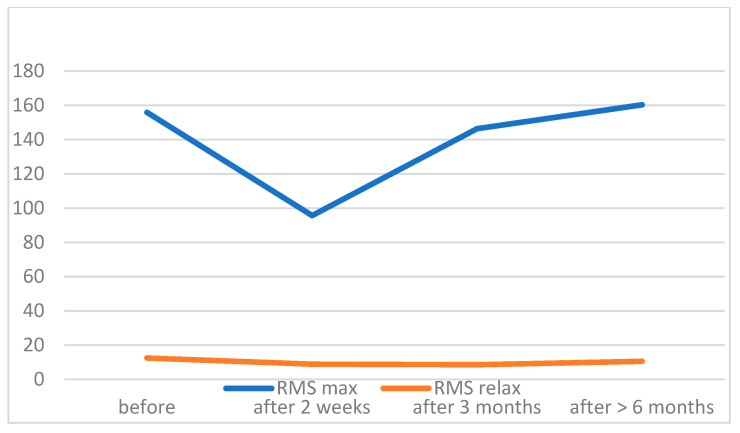
Observation of the RMS values in ptotic patients before and after surgery (mean values).

During maximal contraction of the OOM in the patient group 2 weeks after surgery, we found significantly lower RMS values with respect to the preoperative results. These values were increased after 3 months; however, they remained significantly lower than before the surgery. After 6 months, we found no statistically significant differences in RMS values compared to preoperative results. RMS values of the OOM in relaxation were similar during the whole period of observation. Table 3 provides data confirming the results visualized in Figure 4.

## 4. Discussion

The presented study aimed to investigate the feasibility of a novel technique of sEMG with the electrode placed in the mid-pretarsal area of the upper eyelid and to follow the OOM recovery after the anterior LAA procedure using that method.

### 4.1. Study Implementation

The experiment showed that it is possible to implement the proposed technique in healthy individuals and patients with ptosis. It may be useful in clinical practice, as it is easy to perform, fast, and repeatable. sEMG has proven to be a valuable method in many research and clinical applications [10,11,12,13]. However, it is not being used in eyelid muscle activity assessment in clinical practice. It is in the field of interest of researchers, as it has strong advantages. It is noninvasive, repeatable, quantitative, and objective [14]. Nevertheless, the muscle crosstalk and artifacts may limit the feasibility of sEMG in the assessment of OOM activity [15]. Therefore, optimal electrode placement is being investigated. The OOM muscle may be arbitrarily divided into the orbital and palpebral parts, with the latter being further divided into the preseptal and pretarsal portions [8,16]. To date, electrode placement around the orbital margin was utilized. Choosing the mid-pretarsal area of the upper eyelid seems reasonable, since the measurement is taken directly from the part of the OOM that has been damaged during surgery. The superomedial quadrant of the orbit has been proposed for facial palsy pacing [4]. The mid-pretarsal area of the upper eyelid is free from the influence of adjacent muscles due to the anatomical conditions and therefore presents less crosstalk. It has been proven to represent the high voltage of the mean blink EMG. Akamatsu et al. evidenced that the mean voltage was highest at the central lower eyelid (449.9 μV), followed by the central upper eyelid (430.8 μV) [5]. Future studies could investigate the differences in RMS values obtained with the electrode placed in the mid-pretarsal region and with electrodes located around the orbital margin.

In our experiment, the electrode position allowed the participants to perform all the movements with no discomfort. There were no difficulties in understanding and correctly performing all the movements. sEMG was regarded as a relatively simple procedure and thus has potential for popularity.

The size of each subject’s face differs. As a result, the location of activated muscle regions in each participant is inconsistent. This is a downside of electrode placement around the orbital margin. In this study, the electrode placement was consistent in each participant in the region occupied only by OOM independently from the face size. That may facilitate the standardization of future studies and make them more comparable.

### 4.2. Comparison between Controls and Patients

To our knowledge, this is the largest group examined with the eyelid sEMG technique. The results revealed that the RMS values of the OOM in healthy subjects were comparable to ptotic patients prior to surgery. This finding indicates that values obtained with this method are reliable based on the group size.

### 4.3. Patients before and after Surgery

Iatrogenic damage of the OOM occurs during many oculoplastic procedures. LAA is a procedure that can be performed through a skin incision or through a conjunctival approach [1,2]. The anterior approach requires an incision in the OOM to gain access to the levator aponeurosis. In many cases, the procedure is combined with blepharoplasty with excision of the strip of the OOM. Reliable preoperative assessment of OOM activity could result in choosing a less-aggressive surgical technique or, when possible, a posterior approach. We found that the OOM suffers from the anterior LAA procedure but mostly recovers within 3 months after the operation. We noticed a statistically significant (*p* < 0.05) decrease in RMS values of maximal contraction of the OOM 2 weeks after surgery. An improvement in muscle activation within the first 3 months after surgery was observed. However, there were still statistically significant differences between OOM activation patterns before surgery and 3 months after (*p* = 0.03). We found no statistical differences between RMS values pre-op and post-op more than 6 months after surgery. Future studies can be conducted to detect the onset of OOM recovery during the early follow-up after surgery. Moreover, it would be interesting to investigate the relationship between OOM electrophysiological recovery and the presence of lagophthalmos.

### 4.4. Advantages and Limitations

These preliminary results provide the basis for further research towards a new approach to OOM activity assessment in clinical practice. The presented technique offers several advantages. It is simple and fast, as placing only one electrode reduces the preparation time and costs. It does not require special training or anatomical knowledge by the technician to properly install the electrodes. Therefore, it is repeatable and can be applied by different technicians with no loss to the quality of data collected. All these advantages can increase the utility of eyelid sEMG in a clinical setting.

The disadvantages of this technique are as follows:

1. The electrodes attached to the upper eyelid may disconnect during forced eyelid closure since the electrodes are secured with adhesive tape. This problem could be resolved by introducing new materials used for electrode construction, such as soft carbons [17].
2. The electrode can be attached exclusively to the undamaged skin. This limits the utility of the procedure within the first week post-op.
3. We encountered some difficulties in correctly placing the electrodes in patients with dermatochalasis. Excessive drooping of the skin may be a contraindication in some cases.

In conclusion, the results of the present study indicate that the OOM recovers from surgical injury within 6 months after the anterior approach LAA procedure. Electrode placement in the mid-pretarsal region of the upper eyelid is a novel approach introduced to limit crosstalk. sEMG may become a useful diagnostic tool in OOM activation assessment in an objective manner. However, further studies are needed to verify the usefulness of eyelid sEMG with the electrode placed in the mid-pretarsal area.

## Figures and Tables

**Table 1:** Demographics of the control group (Controls) and ptotic patients (Patients).

Variables	Controls (*n* = 39)		Patients (*n* = 29)		
Gender (F/M)	26/13		24/5		
	Mean ± SD	Min–Max	Mean ± SD	Min–Max	*p*-Value
Age (years)	67.28 ± 12.8	39–87	67.16 ± 83	45–83	0.96
Age F (years)	66.85 ± 12.54	40–87	65.81 ± 83	45–83	0.78
Age M (years)	68.15 ± 13.79	39–86	70.4 ± 78	64–78	0.73
Body height (cm)	168 ± 33.19	154–189	166 ± 183	156–183	0.89

F: female; M: male.

**Table 2:** Comparison of the OOM RMS values in maximal contraction (RMS max) and relaxation (RMS relax) between control group and ptotic patients.

Variables	Controls (*n* = 39)				Patients (*n* = 29)				
	Mean ± SD		Min–Max		Mean ± SD		Min–Max		*p*-Value
	RMS Max	RMS Relax	RMS Max	RMS Relax	RMS Max	RMS Relax	RMS Max	RMS Relax	
RMS	150.1 ± 84.65	10.21 ± 6.82	14.7–321.3	3.2–35.3	160.37 ± 74.52	11.84 ± 5.18	64.8–239.3	64.8–25.7	0.85
Right eye	149.44 ± 85.91	10.51 ± 4.5	14.7–312	3.3–21.7	160.38 ± 85.55	13.06 ± 5.72	45–305.3	45–25.7	0.73
Left eye	150.77 ± 83.38	9.92 ± 8.5	64.6–255.7	4.1–35.3	148.18 ± 87.98	10.62 ± 4.23	55.4–243.7	55.4–16.7	0.94
Male	163.8 ± 88.15	7.45 ± 3.08	87.1–249.3	17.1–35.3	141.08 ± 76.11	13,12 ± 3.33	93.3–189.3	93.3–18.9	0.57
Female	143.25 ± 82.34	11.6 ± 7.97	14.7–321.3	3.2–35.3	162.25 ± 88.4	11.65 ± 5.37	45–239.3	45–25.7	0.57
Right eye male	171.67 ± 92.79	11.45 ± 3.67	96–249.3	3.3–17.1	145.86 ± 71.62	15.23 ± 2.98	93.3–189	93.3–18.9	0.4
Right eye female	138.33 ± 81.4	6.27 ± 4.79	14.7–209.7	6–21.7	164.64 ± 86.92	12.74 ± 5.96	71.9–239.3	71.9–25.7	0.69
Left eye male	155.94 ± 82.94	6.27 ± 2.17	87.1–249	4.1–9.7	136.3 ± 75.02	11 ± 2.1	93.8–189.3	93.8–13	0.21
Left eye female	148.18 ± 83.2	11.75 ± 10.11	64.6–321.3	3.2–35.3	159.85 ± 89.82	10.56 ± 4.46	45–305.3	45–16.7	0.7

**Table 3:** Summary of the RMS values in patient group at certain periods of observation.

	Mean ± SD		Min–Max		*p*-Value	
	RMS Max	RMS Relax	RMS Max	RMS Relax	RMS Max	RMS Relax
before	160.37 ± 74.52	11.84 ± 5.18	64.8–239.3	4.7–25.7	N.A. *	N.A. *
2 weeks	125.2 ± 54.04	9.8 ± 4.45	8–266	5–19.9	<0.00001	0.0158
3 months	154.68 ± 46.63	10.38 ± 4.88	55.4–213	4.9–22.5	0.00362	0.01314
6 months	161.2 ± 27.99	12.19 ± 4.45	111–210.3	4.2–18.9	0.08364	0.97606

* N.A.: non aplicable.

## Data Availability

Data are available upon reasonable request.
